# 2,2′-Dimethoxy­biphen­yl

**DOI:** 10.1107/S1600536808025178

**Published:** 2008-08-09

**Authors:** Kosuke Nakaema, Akiko Okamoto, Satoshi Maruyama, Keiichi Noguchi, Noriyuki Yonezawa

**Affiliations:** a Department of Organic and Polymer Materials Chemistry, Tokyo University of Agriculture & Technology, Koganei, Tokyo 184-8588, Japan; b TDK Corporation, 2-15-7, Hiagashi Owada, Ichikawa, Chiba 272-8558, Japan; c Instrumentaion Analysis Center, Tokyo University of Agriculture & Technology, Koganei, Tokyo 184-8588, Japan

## Abstract

The mol­ecule of the title compound, C_14_H_14_O_2_, lies on a crystallographic twofold axis perpendicular to the central C—C bond; there is one half-mol­ecule in the asymmetric unit. The angle between the least-squares planes of the two aromatic rings is 66.94 (7)°. The meth­oxy group, with a twist angle of 10.69 (8)°, is slightly out of the plane of the benzene ring. In the crystal structure, C—H⋯π inter­actions are observed between adjacent mol­ecules along the *c*-axis direction.

## Related literature

For related literature, see: Hargreaves *et al.* (1961 ▶); Yonezawa *et al.* (1993 ▶, 2000 ▶, 2003 ▶); Iyoda *et al.* (1990 ▶).
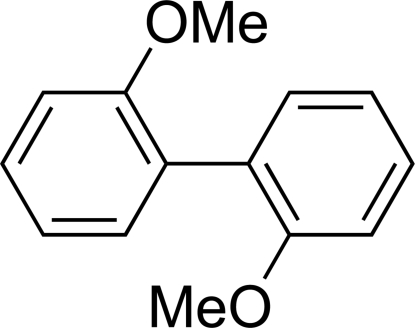


## Experimental

### 

#### Crystal data

C_14_H_14_O_2_
*M* *_r_* = 214.25
Tetragonal, 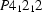

*a* = 7.39307 (13) Å
*c* = 20.1623 (4) Å
*V* = 1102.02 (4) Å^3^
*Z* = 4
Cu *K*α radiation
μ = 0.68 mm^−1^
*T* = 193 K
0.40 × 0.20 × 0.10 mm

#### Data collection

Rigaku R-AXIS RAPID diffractometer
Absorption correction: numerical (*NUMABS*; Higashi, 1999 ▶) *T*_min_ = 0.813, *T*_max_ = 0.934
20356 measured reflections
651 independent reflections
640 reflections with *I* > 2σ(*I*)
*R*_int_ = 0.024

#### Refinement

*R*[*F* ^2^ > 2σ(*F* ^2^)] = 0.024
*wR*(*F* ^2^) = 0.071
*S* = 1.14
651 reflections
75 parameters
H-atom parameters constrained
Δρ_max_ = 0.15 e Å^−3^
Δρ_min_ = −0.11 e Å^−3^

### 

Data collection: *PROCESS-AUTO* (Rigaku, 1998 ▶); cell refinement: *PROCESS-AUTO*; data reduction: *CrystalStructure* (Rigaku/MSC, 2004 ▶); program(s) used to solve structure: *SIR2004* (Burla *et al.*, 2005 ▶); program(s) used to refine structure: *SHELXL97* (Sheldrick, 2008 ▶); molecular graphics: *ORTEPIII* (Burnett & Johnson, 1996 ▶); software used to prepare material for publication: *SHELXL97*.

## Supplementary Material

Crystal structure: contains datablocks global, I. DOI: 10.1107/S1600536808025178/fl2209sup1.cif

Structure factors: contains datablocks I. DOI: 10.1107/S1600536808025178/fl2209Isup2.hkl

Additional supplementary materials:  crystallographic information; 3D view; checkCIF report

## Figures and Tables

**Table 1:** Hydrogen-bond geometry (Å, °)

*D*—H⋯*A*	*D*—H	H⋯*A*	*D*⋯*A*	*D*—H⋯*A*
C3—H3⋯*Cg*1^i^	0.95	2.85	3.7266 (14)	154

Symmetry code: (i) 
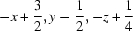
. *Cg*1 is the centroid of atoms C1–C6.

**Fig. 1.**
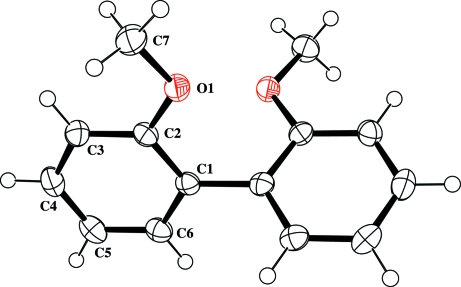
Molecular structure of (I), with the atom-labeling scheme and displacement ellipsoids drawn at the 50% probability level. Unlabeled atoms are related to labeled atoms by x, y, -z.

**Fig. 2.**
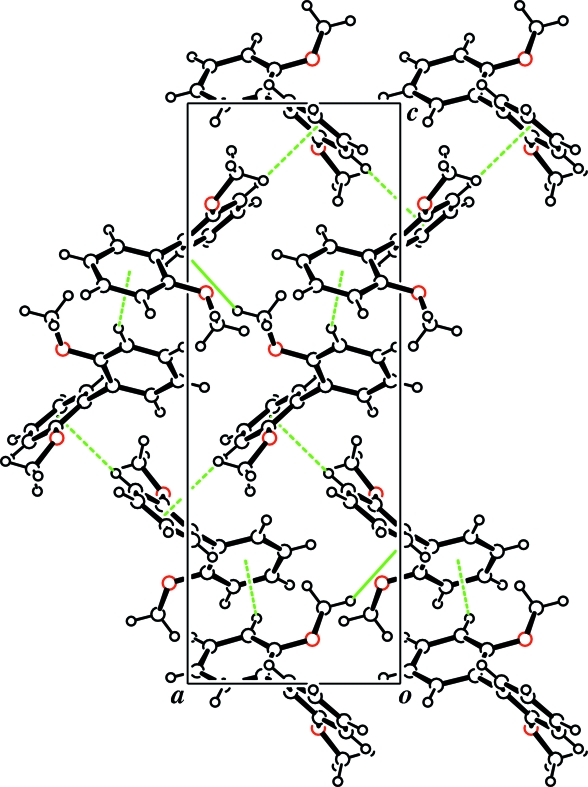
A partial packing diagram of (I), viewed down the b-axis. Green broken and full lines indicate the C—H···π and short C···H interactions, respectively.

## supplementary crystallographic information

### Comment

Biphenyl is the simplest example of aromatic ring assemblies in which aromatic rings are connected by a single bond. The apparent single bond  lies behind the characteristic diversity in the molecular structure and chemical properties of these assemblies. As with the formal single bonding of alternative polyenes, the single bond  between the aromatic rings  has the nature of double bonding. However, the steric hindrance of  substituents, especially at the *o*-positions,  collapses  coplanarity lowering the stabilizing conjugative effects on the structure. The non-substituted biphenyl is planar in the solid state in spite of the *o*-protons' repulsion (Hargreaves *et al.*, 1961) but it has a twisted conformation in solution. In the past decade and a half, the authors have demonstrated the excellent acyl-accepting ability of (I), an *o*,*o'*-disubstituted biphenyl, in consecutive dual electrophilic aromatic aroylation reactions, especially in condensation polymerization or monomer preparation for wholly aromatic polyketones (Yonezawa *et al.*, 1993, 2000, 2003). The strong electron donating ability of the *o*-methoxy group should make (I) highly reactive against electrophiles not only for a first acylation but also for a second one, even though the introduced ketonic carbonyl group has a strong electron-withdrawing nature. The maintenance of high reactivity after the first acylation is thought to be brought about by the suitable conformation of the mono-acylated intermediate preventing the transmission of the electron-withdrawing effect of the acyl group to the other aromatic ring.

The molecule of (I) lies across a crystallographic 2-fold axis so that the asymmetric unit contains one-half  molecule (Fig. 1). The 2-fold axis is perpendicular to the central C—C bond of (I). The angle between the least-squares planes of the two phenyl rings is 66.94 (7)°. The methoxy group is slightly out of the plane of the phenyl ring with the angle between the O1—C7 bond and least-squares plane of the phenyl ring at 10.69 (8)°. The molecular packing of (I) is mainly stabilized by van der Waals interactions. In addition, C—H···π interactions are observed between  adjacent molecules along the *c*-direction (Fig. 2). The distance between H3 and *Cg* (the ring center of gravity) at (3/2 - *x*, *y* - 1/2, 1/4 - *z*) is 2.85 Å. The slightly short intermolecular C6···H7A at (*y* + 1/2, 1/2 - *x*, *z* - 1/4) contact of 2.86 Å is also found.

### Experimental

To the solution of 2,2'-dihydroxybiphenyl (5.5 g, 26 mmol) in an aqueous NaOH solution (3.2 wt-%, 220 ml), dimethyl sulfate (16 g, 127 mmol) was added dropwise over a period of 10 min with ice-cooling and vigorous stirring. After stirring for 3 h, the resulting precipitate was collected by filtration and dissolved in chloroform (*ca* 100 ml). The solution was washed with aqueous 1 *M* NaOH solution (*ca* 100 ml) and dried over granular magnesium sulfate. The crude product obtained by evaporation of the above solution was recrystallized from acetone. Yield 72%. *M*.p. 154–154.5°C. Lit. 154–155°C(Iyoda *et al.*, 1990). Colorless single crystals suitable for X-ray diffraction were obtained by simple standing of the hot acetone solution of the crude crystals at room temperature in an Erlenmeyer flask with a cork-stopper.

### Refinement

All the H atoms were found in difference maps and were subsequently refined as riding atoms, with C—H = 0.95 (aromatic) and 0.98 (methyl) Å, and *U*ĩso~(H) = 1.2*U*~eq~(C). The anomalous scattering signal of (I) is too weak to predict the accurate absolute structure. Therefore, the merging of Friedel-pair data was performed before final refinement.

### Crystal data

**Table:**

C_14_H_14_O_2_	*Z* = 4
*M**_r_* = 214.25	*F*_000_ = 456
Tetragonal, *P*4_1_2_1_2	*D*_x_ = 1.291 Mg m^−^^3^
Hall symbol: P 4abw 2nw	Melting point = 427.0–427.5 K
*a* = 7.39307 (13) Å	Cu *K*α radiation λ = 1.54187 Å
*b* = 7.39307 (13) Å	Cell parameters from 19640 reflections
*c* = 20.1623 (4) Å	θ = 4.4–68.2º
α = 90º	µ = 0.68 mm^−^^1^
β = 90º	*T* = 193 K
γ = 90º	Block, colorless
*V* = 1102.02 (4) Å^3^	0.40 × 0.20 × 0.10 mm

### Data collection

**Table:**

Rigaku R-AXIS RAPID diffractometer	651 independent reflections
Radiation source: rotating anode	640 reflections with *I* > 2σ(*I*)
Monochromator: graphite	*R*_int_ = 0.024
Detector resolution: 10.00 pixels mm^-1^	θ_max_ = 68.2º
*T* = 193 K	θ_min_ = 6.4º
ω scans	*h* = −8→8
Absorption correction: numerical(NUMABS; Higashi, 1999)	*k* = −8→8
*T*_min_ = 0.813, *T*_max_ = 0.934	*l* = −24→24
20356 measured reflections	

### Refinement

**Table:**

Refinement on *F*^2^	Hydrogen site location: difference Fourier map
Least-squares matrix: full	H-atom parameters constrained
*R*[*F*^2^ > 2σ(*F*^2^)] = 0.024	*w* = 1/[σ^2^(*F*_o_^2^) + (0.0428*P*)^2^ + 0.1367*P*] where *P* = (*F*_o_^2^ + 2*F*_c_^2^)/3
*wR*(*F*^2^) = 0.071	(Δ/σ)_max_ < 0.001
*S* = 1.14	Δρ_max_ = 0.15 e Å^−^^3^
651 reflections	Δρ_min_ = −0.11 e Å^−^^3^
75 parameters	Extinction correction: SHELXL97 (Sheldrick, 2008), Fc^*^=kFc[1+0.001xFc^2^λ^3^/sin(2θ)]^-1/4^
Primary atom site location: structure-invariant direct methods	Extinction coefficient: 0.0079 (11)
Secondary atom site location: difference Fourier map	

### Special details

**Table:**

Geometry. All e.s.d.'s (except the e.s.d. in the dihedral angle between two l.s. planes) are estimated using the full covariance matrix. The cell e.s.d.'s are taken into account individually in the estimation of e.s.d.'s in distances, angles and torsion angles; correlations between e.s.d.'s in cell parameters are only used when they are defined by crystal symmetry. An approximate (isotropic) treatment of cell e.s.d.'s is used for estimating e.s.d.'s involving l.s. planes.
Refinement. Refinement of *F*^2^ against ALL reflections. The weighted *R*-factor *wR* and goodness of fit *S* are based on *F*^2^, conventional *R*-factors *R* are based on *F*, with *F* set to zero for negative *F*^2^. The threshold expression of *F*^2^ > σ(*F*^2^) is used only for calculating *R*-factors(gt) *etc*. and is not relevant to the choice of reflections for refinement. *R*-factors based on *F*^2^ are statistically about twice as large as those based on *F*, and *R*- factors based on ALL data will be even larger.

### Fractional atomic coordinates and isotropic or equivalent isotropic displacement parameters (Å^2^)

**Table:**

	*x*	*y*	*z*	*U*_iso_*/*U*_eq_	
O1	0.41372 (12)	0.38528 (13)	0.07663 (4)	0.0307 (3)	
C1	0.64788 (17)	0.51523 (17)	0.01376 (5)	0.0237 (3)	
C2	0.59201 (17)	0.38066 (18)	0.05852 (6)	0.0249 (3)	
C3	0.71465 (19)	0.25505 (19)	0.08287 (6)	0.0297 (3)	
H3	0.6753	0.1627	0.1123	0.036*	
C4	0.89583 (18)	0.2648 (2)	0.06399 (7)	0.0332 (4)	
H4	0.9793	0.1774	0.0800	0.040*	
C5	0.95499 (19)	0.4005 (2)	0.02208 (6)	0.0338 (4)	
H5	1.0792	0.4093	0.0105	0.041*	
C6	0.83034 (18)	0.52435 (19)	−0.00291 (6)	0.0290 (3)	
H6	0.8708	0.6171	−0.0320	0.035*	
C7	0.3605 (2)	0.2711 (2)	0.13032 (7)	0.0364 (4)	
H7A	0.2347	0.2969	0.1423	0.044*	
H7B	0.4388	0.2939	0.1686	0.044*	
H7C	0.3715	0.1442	0.1169	0.044*	

### Atomic displacement parameters (Å^2^)

**Table:**

	*U*^11^	*U*^22^	*U*^33^	*U*^12^	*U*^13^	*U*^23^
O1	0.0242 (5)	0.0351 (6)	0.0327 (5)	0.0000 (4)	0.0018 (4)	0.0101 (4)
C1	0.0259 (7)	0.0252 (7)	0.0201 (6)	−0.0028 (5)	−0.0028 (5)	−0.0028 (5)
C2	0.0244 (6)	0.0274 (7)	0.0229 (6)	−0.0016 (5)	−0.0030 (5)	−0.0025 (5)
C3	0.0327 (7)	0.0281 (7)	0.0282 (6)	−0.0012 (6)	−0.0051 (5)	0.0031 (6)
C4	0.0299 (7)	0.0359 (8)	0.0339 (7)	0.0067 (6)	−0.0073 (6)	−0.0019 (6)
C5	0.0238 (7)	0.0462 (9)	0.0313 (7)	0.0004 (6)	−0.0014 (5)	−0.0038 (6)
C6	0.0284 (7)	0.0350 (8)	0.0237 (6)	−0.0048 (6)	−0.0004 (6)	−0.0009 (6)
C7	0.0351 (8)	0.0410 (9)	0.0331 (6)	−0.0020 (6)	0.0055 (6)	0.0094 (6)

### Geometric parameters (Å, °)

**Table:**

O1—C2	1.3682 (16)	C4—C5	1.383 (2)
O1—C7	1.4279 (16)	C4—H4	0.9500
C1—C6	1.3918 (18)	C5—C6	1.393 (2)
C1—C2	1.4053 (18)	C5—H5	0.9500
C1—C1^i^	1.494 (3)	C6—H6	0.9500
C2—C3	1.3876 (18)	C7—H7A	0.9800
C3—C4	1.3944 (19)	C7—H7B	0.9800
C3—H3	0.9500	C7—H7C	0.9800
			
C2—O1—C7	116.93 (11)	C4—C5—C6	119.25 (14)
C6—C1—C2	118.31 (12)	C4—C5—H5	120.4
C6—C1—C1^i^	120.99 (10)	C6—C5—H5	120.4
C2—C1—C1^i^	120.69 (10)	C1—C6—C5	121.46 (13)
O1—C2—C3	123.49 (12)	C1—C6—H6	119.3
O1—C2—C1	115.90 (11)	C5—C6—H6	119.3
C3—C2—C1	120.60 (12)	O1—C7—H7A	109.5
C2—C3—C4	119.78 (13)	O1—C7—H7B	109.5
C2—C3—H3	120.1	H7A—C7—H7B	109.5
C4—C3—H3	120.1	O1—C7—H7C	109.5
C5—C4—C3	120.53 (13)	H7A—C7—H7C	109.5
C5—C4—H4	119.7	H7B—C7—H7C	109.5
C3—C4—H4	119.7		
			
C7—O1—C2—C3	9.30 (18)	C1—C2—C3—C4	1.5 (2)
C7—O1—C2—C1	−169.45 (11)	C2—C3—C4—C5	1.1 (2)
C6—C1—C2—O1	175.77 (11)	C3—C4—C5—C6	−2.1 (2)
C1^i^—C1—C2—O1	−2.66 (18)	C2—C1—C6—C5	2.0 (2)
C6—C1—C2—C3	−3.02 (18)	C1^i^—C1—C6—C5	−179.53 (12)
C1^i^—C1—C2—C3	178.54 (12)	C4—C5—C6—C1	0.5 (2)
O1—C2—C3—C4	−177.22 (12)		

Symmetry codes: (i) *y*, *x*, −*z*.

### Hydrogen-bond geometry (Å, °)

**Table:**

*D*—H···*A*	*D*—H	H···*A*	*D*···*A*	*D*—H···*A*
C3—H3···Cg1^ii^	0.95	2.85	3.7266 (14)	154

Symmetry codes: (ii) −*x*+3/2, *y*−1/2, −*z*+1/4.
